# Tolerability of N-chlorotaurine in comparison with routinely used antiseptics: an in vitro study on chondrocytes

**DOI:** 10.1007/s43440-024-00601-9

**Published:** 2024-05-17

**Authors:** Magdalena Pilz, Kevin Staats, Ojan Assadian, Reinhard Windhager, Johannes Holinka

**Affiliations:** 1 Division of Orthopaedics, Department of Orthopaedics and Trauma Surgery, Medical University of Vienna, Waehringer Guertel 18-20, 1090 Vienna, Austria; 2 Regional Hospital Wiener Neustadt, Corvinusring 3-5, 2700 Wiener Neustadt, Lower Austria Austria; 3 Present Address: Department of Dermatology, Medical University of Vienna, Waehringer Guertel 18-20, 1090 Vienna, Austria

**Keywords:** Antiseptics, Chondrocytes, Cytotoxicity, N-Chlorotaurine, Septic joint infection

## Abstract

**Background:**

Currently, povidone-iodine (PVP-I) and hydrogen peroxide (H_2_O_2_) are frequently used antiseptics in joint infections, but the cytotoxic effects of these solutions are already reported. N-chlorotaurine (NCT) shows a broad-spectrum bactericidal activity and is well tolerated in various tissues, but its effect on human chondrocytes is unknown. The purpose of this study was to assess the cytotoxic effect of NCT, PVP-I, and H_2_O_2_ on human chondrocytes compared to a control group in an in vitro setting to get first indications if NCT might be a promising antiseptic in the treatment of septic joint infections for the future.

**Material and methods:**

Chondrocytes extracted from human cartilage were incubated with various concentrations of NCT, PVP-I, and H_2_O_2_ for 5 and 30 min respectively. EZ4U cell viability kit was used according to the manufacturer’s recommendations determining cell viability. To assess cell viability based on their nuclear morphology, cells were stained with acridine-orange and identified under the fluorescence microscope.

**Results:**

EZ4U kit showed after 5 and 30 min of incubation a significant decrease in cell viability at NCT 1%, NCT 0.1%, PVP-I, and H_2_O_2_, but not for NCT 0.001% and NCT 0.01%. Acridine-orange staining likewise presented a significant decrease in vital cells for all tested solutions except NCT 0.001% and NCT 0.01% after 5 and 30 min of incubation.

**Conclusion:**

Our results demonstrate that NCT is well tolerated by chondrocytes in vitro at the tested lower NCT concentrations 0.01% and 0.001% in contrast to the higher NCT concentrations 1% and 0.1%, PVP-I (1.1%), and H_2_O_2_ (3%), for which a significant decrease in cell viability was detected. Considering that the in vivo tolerability is usually significantly higher, our findings could be an indication that cartilage tissue in vivo would tolerate the already clinically used 1% NCT solution. In combination with the broad-spectrum bactericidal activity, NCT may be a promising antiseptic for the treatment of septic joint infections.

**Graphical abstract:**

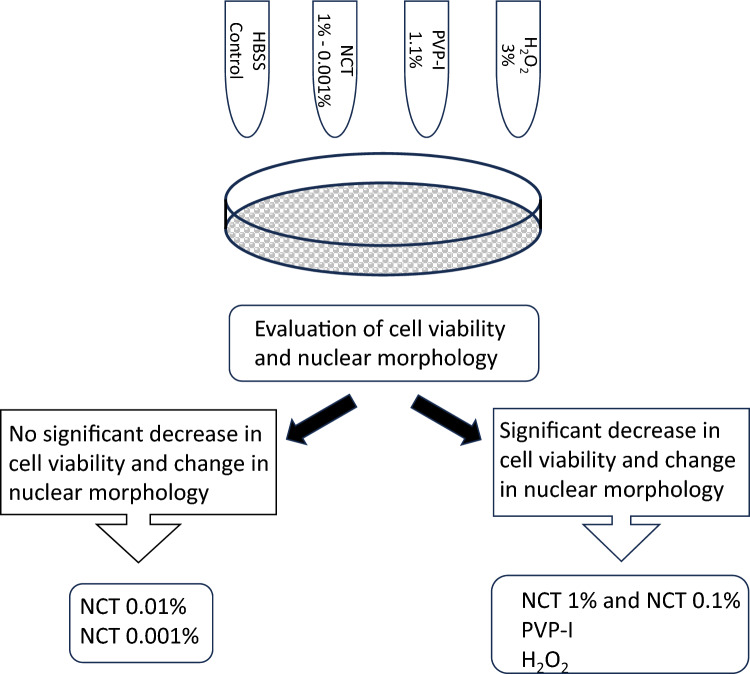

## Introduction

Septic joint infections are rare occurrences [1] with an estimated global incidence of between six and ten events per 100,000 individuals per year [2], but inadequate treatment may lead to an irreversible erosion of the joint and subsequently disability due to secondary arthritis [3]. The etiology of septic joint infections varies, the most common reason is hematogenous spreading during a bacteremic episode [1]. Pathogens, responsible for acute septic arthritis, may also penetrate directly during iatrogenic procedures like arthrocentesis or arthroscopy or in the course of a trauma. The most frequently found pathogen in septic joint infections is *Staphylococcus aureus*, followed by streptococci [4, 5].

The intraoperative administration and instillation of local antiseptics in joint infections is a commonly used clinical practice, however, there is an ongoing debate about the applicable compounds, since possible cytotoxicity may cause additional damage to articular cartilage [6–8].

Currently, a frequently used antiseptic in orthopedic surgery is povidone-iodine (PVP-I) [6, 7, 9], due to its more favorable tolerability in cartilage cells compared to other antiseptics such as Octenidine-dihydrochloride (OCT) or polyhexanide (PHMB) [10]. However, experimentally conducted animal studies reported a cytotoxic effect of PVP-I on articular cartilage and synovial cells of rats [11] and bovine superficial cartilage layers when administered longer than one minute [9]. A further frequently used antiseptic in local joint and wound infections is hydrogen peroxide (H_2_O_2_) [8], which, at least for higher concentrations, negatively influences human dermal fibroblasts by reducing their proliferation and migration [12] and proteoglycan synthesis resulting in a loss of chondrocytes and cartilage damage [8].

Taurine, a sulfur-containing amino acid, is present in high concentrations in leukocytes [13] and performs an important part in various biological processes, including immunity, reproduction, membrane stabilization, calcium modulation, and development of the central nervous system [14]. It eliminates hypochlorous acid, an effective oxidant, resulting in N-chlorotaurine (NCT) [13]. The broad-spectrum bactericidal activity of NCT on various bacterial strains including *Staphylococcus aureus* and *Streptococcus pyogenes* has already been proven [15, 17], even against multidrug-resistant test strains [18]. In addition, Nagl et al. [16] reported a pronounced post-antibiotic effect of NCT in a mouse peritonitis model.

Besides the bactericidal activity of NCT, anti-inflammatory properties by inhibiting the production of reactive oxygen species and pro-inflammatory cytokines have been reported. This fact is very interesting for the treatment of multi-factorial diseases like chronic periodontitis, which is the result of a complex interaction between bacteria and the inflammatory response of the host [19].

The tolerability of this potent antiseptic has been reported previously for various body sites like the ear, skin, eye, mucous membranes, and the urinary bladder [20].

However, so far, the effects of NCT on human cartilage tissue remain unknown. So, it is an important step to get first indications, if NCT in adequate concentrations would also be tolerated by human chondrocytes. In this first evaluation of the tolerability of NCT, the dose- and time-dependent effects of the antiseptics NCT, PVP-I, and H_2_O_2_ on human chondrocytes have been determined. The aim of this study was to collect further information about their in vitro cytotoxicity by evaluating the cell viability and nuclear morphology of human chondrocytes in an in vitro setting.

## Materials and methods

### Compliance with ethical standards

Ethical approval: All procedures performed in this study involving human participants were by the ethical standards of the institutional and/or national research committee and with the 1964 Helsinki Declaration and its later amendments or comparable ethical standards. Approval of the Ethics Committee of the Medical University of Vienna was obtained (ethic committee vote no.: 413/2006).

Informed consent: Informed consent was obtained from all participants included in the study.

### Antiseptics

NCT was produced synthetically at the Innsbruck Medical University (Innsbruck, Austria) in the form of a crystalline sodium salt. The molecular weight of the stock solution is 181.57 g/Mol. For the preparation of the test solutions, it was dissolved in Hank’s Balanced Salt Solution (“HBSS”, GIBCO, Grand Island, NY, USA, product number 14025092). The tested concentrations in this study were 1%, 0.1%, 0.01%, and 0.001% (55 mM–55 µM), which were stored in the refrigerator at + 4 to + 8 °C.

Povidone-iodine solution (Mundipharma, Limburg, Germany, pharma central number 01970433) is a commercially available antiseptic. The solution contains 10 g povidone-iodine (poly-vinylpyrrolidone-iodine-complex, PVP-I) per 100 ml, with a total content of 1.1% available iodine.

Hydrogen peroxide solution 3%, in H_2_O, (Gespag, LKH Apotheke, 4820 Bad Ischl, Austria, pharma central number 04652521) is also a commercially available antiseptic, which was used following the manufacturer's recommendations for irrigation.

### Chondrocyte isolation and culture

Patients with a positive history of joint infection, systemic diseases, intra-articular injections less than a year before, hemarthrosis, or smoking were excluded from this study. Cartilage material was dissected aseptically from eight patients undergoing total knee replacement and rinsed in phosphate-buffered saline (PBS, Gibco, Grand Island, NY, USA, product number 10010023).

Cartilage slices from the non-weight bearing area of the femoral condyles were cut into small pieces (1 mm × 1 mm) and digested in 8 ml 100 U/ml collagenase type II (Gibco, Grand Island, NY, USA, product number 17101015) for 24 h in a petri dish (100 mm × 15 mm, BD Falcon™, BD Biosciences, NC, USA) at 37 °C. Afterwards, the material was filtered using a 40 µm cell strainer (BD Falcon™, BD Biosciences, NC, USA). The filtrate was supplemented with 5 ml DMEM-F12 + GlutaMAX (Gibco, Grand Island, NY, USA, product number 31331093) and centrifuged at 1200 rpm for 10 min. The supernatant was discharged, and the cell pellet dissolved in Dulbecco's modified Eagle's medium DMEM/F-12 supplemented with 10% fetal calf serum (FCS; PAA, Pasching, Austria, product number A11-151), 50 µg/ml ascorbic acid (Sigma-Aldrich, MO, USA, product number A5960100G), 200 U/ml penicillin/streptomycin combination and 2.5 µg/ml amphotericin B (Sigma-Aldrich, St. Louis, MO, product number P4333 and A2942) and transferred into a tissue culture flask (BD Falcon™, BD Biosciences, NC, USA). The cells were cultured under standard conditions (37 °C, 95% rH, 5% CO_2_), and the medium was changed twice a week. Approaching 95% confluence, chondrocytes were detached by treatment with trypsin–EDTA (0.25% trypsin/2.21 mM EDTA, Sigma-Aldrich, St. Louis, MO, product number T4049) and sub-cultured. During this process, cells were counted, and vitality was assessed using the trypan blue method, indicating that in all chondrocyte cultures, more than 95% of the cells were vital.

### EZ4U cell viability assay

The EZ4U cell viability kit was used according to the manufacturer’s recommendations (“EZ4U”, Biomedica Group, Vienna, Austria, product number BI-5000). Each well of a flat-bottom 96-well plate was filled with 1 × 10^4^ chondrocytes in 200 µl cell culture medium, which were allowed to adhere for 48 h. Subsequently, the cells were rinsed twice with PBS and incubated at 37 °C in humidified air with various concentrations of NCT (1–0.001%), PVP-I (1.1%) and H_2_O_2_ (3%), each solution in triplets for 5 and 30 min, respectively. The control group was incubated with HBSS. Afterward, cells were rinsed again two times with PBS, each well was filled with 20 µl of EZ4U-substrate-solution and 200 µl HBSS and incubated for two hours. The measurement of the absorbance was performed with a micro-plate reader (Bio-Rad, Model 550, Bio-Rad Laboratories, Hercules, CA, USA) at 450 nm wavelength and 620 nm as reference.

### Acridine-orange fluorescence microscopy

Nuclear morphology was assessed by acridine-orange staining. For this purpose, 5 × 10^4^ cells in 1 ml cell culture medium per well were plated in 24-well plates and let to attach for 48 h. Thereafter, cells were washed two times with PBS and incubated for 5 and 30 min, respectively, with the different concentrations of NCT (1–0.001%), PVP-I (1.1%), H_2_O_2_ (3%), and HBSS in the control group. Following incubation chondrocytes were washed twice with PBS and incubated with 1 ml 1.5% acridine-orange solution (Fluka, Sigma-Aldrich, St. Louis, MO, USA, product number A9231) for 20 min under protection from direct light exposure. Subsequently, cells were rinsed as mentioned above and covered with 1 ml HBSS. For the calculation of the decrease in vital cells, 2 × 100 chondrocytes were counted under the fluorescence microscope (IMT-2, Olympus, Hamburg, Germany) differentiating between vital and dead cells based on their nuclear morphology.

### Statistical analysis

The data from our experiments were analysed using the Kruskal–Wallis test for nonparametric data and Dunn’s test for post-hoc analysis. Results were presented as median values with interquartile ranges. A value of p ≤ 0.05 was considered significant. PRISM (Graph Pad, San Diego, CA, USA) version 10.0 for Macintosh was used for statistical analysis.

## Results

### Dose- and time-dependent decrease in cell viability

**Fig. 1:**
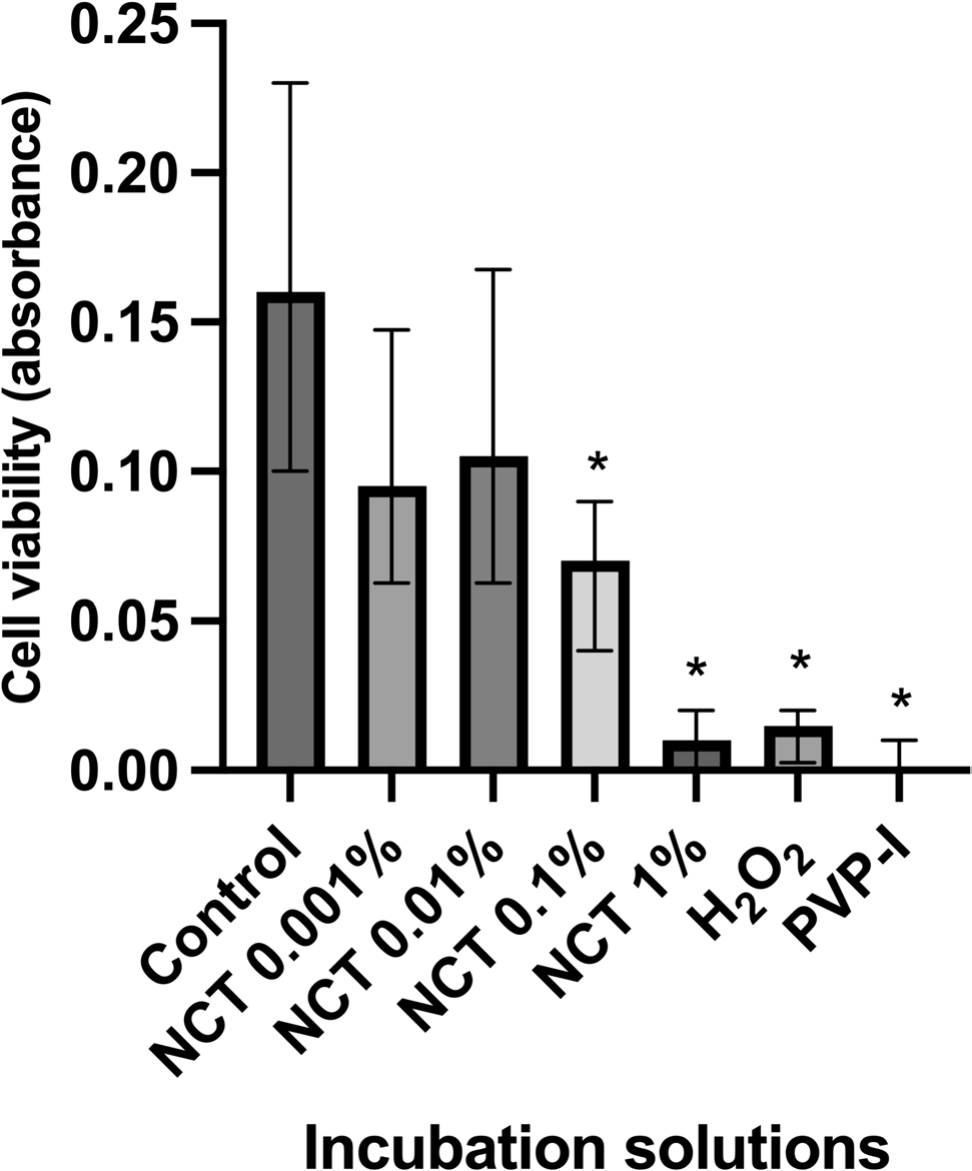
Effect of antiseptics compared to the control group in Hank’s Balanced Salt Solution (HBSS) on cell viability of chondrocytes after 5 min of incubation with N-chlorotaurine (NCT) 1%, NCT 0.1%, NCT 0.01%, NCT 0.001%, povidone-iodine (PVP-I) 1.1%, and hydrogen peroxide (H_2_O_2_) 3%, measured with EZ4U cell viability test. Results were presented as median values with interquartile ranges (* significant value, p ≤ 0.05); Kruskal–Wallis test for nonparametric data, Dunn’s test as post-hoc test. The Kruskal–Wallis test demonstrated a significant decrease in cell viability (H = 125.1, p < 0.0001, N1–N7 = 24). The Dunn’s post-hoc test showed significant results for NCT 1% (p < 0.0001), NCT 0.1% (p = 0.0070), PVP-I 1.1% (p < 0.0001), and H_2_O_2_ 3% (p < 0.0001). Only for NCT 0.001% (p = 0.8102) and NCT 0.01% (p > 0.9999) decrease of cell viability was not significant

Cell viability was measured using the EZ4U kit after 5 min respectively 30 min of incubation. After 5 min of incubation the Kruskal -Wallis test showed a significant decrease in cell viability (H = 125.1, p < 0.0001, N1–N7 = 24). The Dunn’s post-hoc test revealed a significant decrease in cell viability at NCT 1% (p < 0.0001), NCT 0.1% (p < 0.0070), PVP-I (1.1%) (p < 0.0001), and H_2_O_2_ (3%) (p < 0.0001), in contrast for NCT 0.001% (p > 0.8102) and NCT 0.01% (p = 0.9999) chondrocytes showed no significant decrease in cell viability compared to the control group (Fig. 1).

**Fig. 2:**
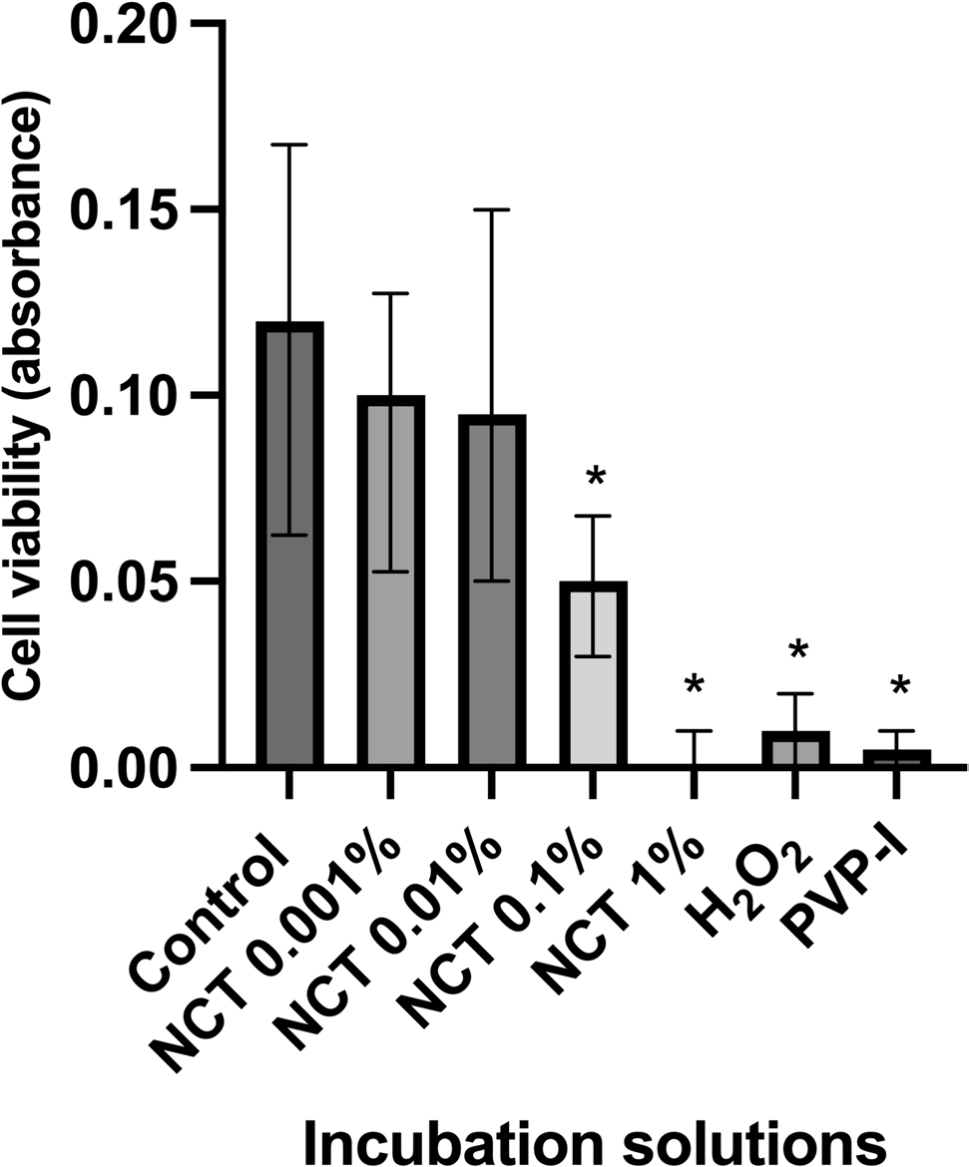
Effect of antiseptics compared to the control group in Hank’s Balanced Salt Solution (HBSS) on cell viability of chondrocytes after 30 min of incubation with N-chlorotaurine (NCT) 1%, NCT 0.1%, NCT 0.01%, NCT 0.001%, povidone-iodine (PVP-I) 1.1%, and hydrogen peroxide (H_2_O_2_) 3%, measured with EZ4U cell viability test. Results were presented as median values with interquartile ranges (* significant value, p ≤ 0.05); Kruskal–Wallis test for nonparametric data, Dunn’s test as post-hoc test. The Kruskal–Wallis test demonstrated a significant decrease in cell viability (H = 123.5, p < 0.0001, N1–N7 = 24). The Dunn’s post-hoc test showed significant results for NCT 1% (p < 0.0001), NCT 0.1% (p = 0.0244), PVP-I 1.1% (p < 0.0001), and H_2_O_2_ 3% (p < 0.0001). Only for NCT 0.001% (p > 0.9999) and NCT 0.01% (p > 0.9999) decrease of cell viability was not significant

After 30 min of incubation again the Kruskal–Wallis test showed a significant decrease in cell viability (H = 123.5, p < 0.0001, N1–N7 = 24). The Dunn’s post-hoc test indicated significant decreases in cell viability for those cultures incubated with NCT 1% (p < 0.0001), NCT 0.1% (p < 0.0244), PVP-I (1.1%) (P < 0.0001), and H_2_O_2_ (3%) (p < 0.0001). As measured before, at NCT 0.001% (p > 0.9999) and NCT 0.01% (p > 0.9999) no significant decrease in cell viability was detectable in comparison with the control group (Fig. 2).

### Assessment of nuclear morphology by acridine-orange staining

**Fig. 3:**
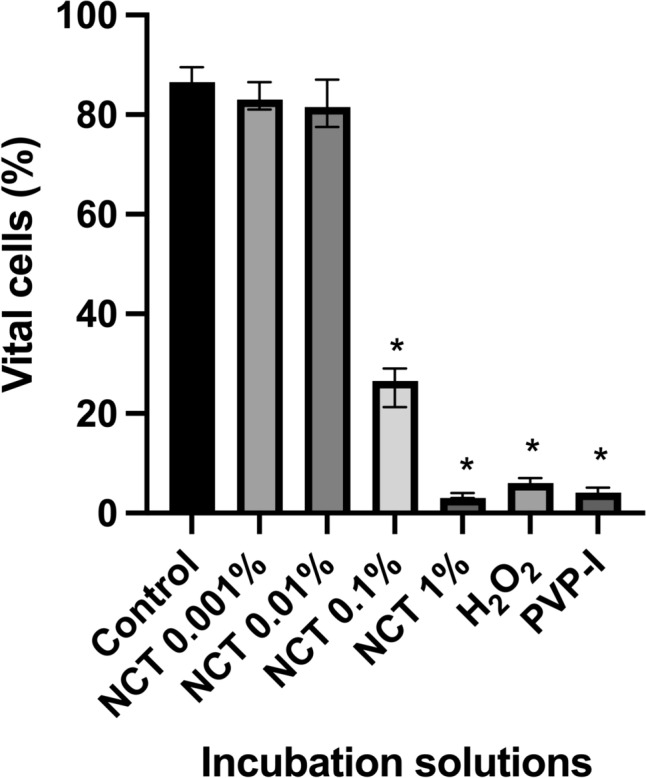
Effect of antiseptics compared to the control group in Hank’s Balanced Salt Solution (HBSS) on nuclear morphology of chondrocytes after 5 min of incubation with N-chlorotaurine (NCT) 1%, NCT 0.1%, NCT 0.01%, NCT 0.001%, povidone-iodine (PVP-I) 1.1%, and hydrogen peroxide (H_2_O_2_) 3% assessed by acridine-orange staining. Results were presented as median values with interquartile ranges (* significant value, p ≤ 0.05); Kruskal–Wallis test for nonparametric data, Dunn’s test as post-hoc test. The Kruskal–Wallis test demonstrated a significant decrease in vital cells (H = 97.76, p < 0.0001, N1–N7 = 16). The Dunn’s post-hoc test revealed a significant number of cells with altered nuclei for NCT 1% (p < 0.0001), NCT 0.1% (p = 0.0052), PVP-I 1.1% (p < 0.0001), and H_2_O_2_ 3% (p < 0.0001). For the other tested solutions NCT 0.01% (p > 0.9999) and NCT 0.001% (p > 0.9999) results were not significant

After 5 min of incubation, the Kruskal–Wallis test presented a significant number of cells with altered nuclei (H = 97.76, p < 0.0001, N1–N7 = 16). In the Dunn’s post-hoc test a significant decrease in vital cells was presented for NCT 1% (p < 0.0001), NCT 0.1% (p < 0.0052), PVP-I (1.1%) (p < 0.0001), and H_2_O_2_ (3%) (p < 0.0001) the detected decrease in vital cells was significant. (Fig. 3). Only NCT 0.001% (p > 0.9999) and NCT 0.01% (p > 0.9999) showed no significant decrease in the level of vital cells compared to the control group.

**Fig. 4:**
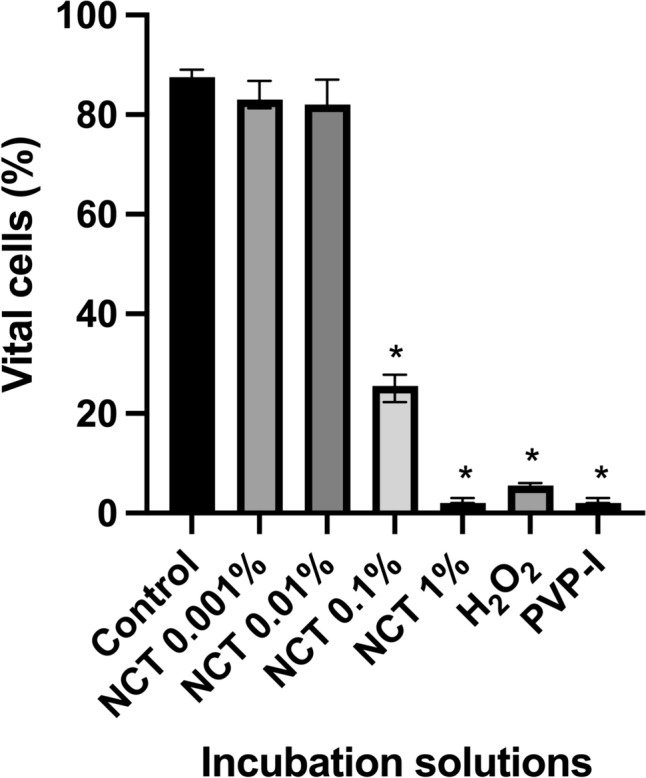
Effect of antiseptics compared to the control group in Hank’s Balanced Salt Solution (HBSS) on cell morphology of chondrocytes after 30 min of incubation with N-chlorotaurine (NCT) 1%, NCT 0.1%, NCT 0.01%, NCT 0.001%, povidone-iodine (PVP-I) 1.1%, and hydrogen peroxide (H_2_O_2_) 3%, assessed by acridine-orange staining. Results were presented as median values with interquartile ranges (* significant value, p ≤ 0.05); Kruskal–Wallis test for nonparametric data, Dunn’s test as post-hoc test. The Kruskal–Wallis test demonstrated a significant decrease in vital cells (H = 100.1, p < 0.0001, N1-N7 = 16). The Dunn’s post-hoc test revealed a significant number of cells with altered nuclei for NCT 1% (p < 0.0001), NCT 0.1% (p = 0.0069), PVP-I 1.1% (p < 0.0001), and H_2_O_2_ 3% (p < 0.0001). For the other tested solutions NCT 0.01% (p > 0.9999) and NCT 0.001% (p > 0.9999) results were not significant

In accordance with the findings after 5 min of incubation, after 30 min of incubation the Kruskal–Wallis test demonstrated a significant decrease in vital cells (H = 100.1, p < 0.0001, N1–N7 = 16). The Dunn’s post-hoc test detected again a significant decrease in vital cells for NCT 1% (p < 0.0001), NCT 0.1% (p = 0.0069), PVP-I (1.1%) (p < 0.0001), and H_2_O_2_ (3%) (p < 0.0001), but not for NCT 0.001% (p > 0.9999) and NCT 0.01% (p > 0.9999) (Fig. 4).

## Discussion

In this study we collected further information about the in vitro cytotoxicity of NCT on human cartilage cells by evaluating the cell viability and nuclear morphology. We demonstrated that NCT-concentrations below 0.1% were well tolerated by chondrocytes compared to higher concentrations of NCT or the other tested antiseptics 1.1% povidone-iodine and 3% H_2_O_2_ solution. The statistical analysis showed that the cell viability did not decrease significantly at the NCT-concentrations 0.001% and 0.01% compared to the control group. At these concentrations the nuclear morphology also did not change in a significant number of cells. So, our results indicate a dose-dependent NCT tolerability in cartilage cells, which has already been shown for different other tissues in various in vitro and in vivo studies [20, 21].

For the treatment of septic joint infections, the application of local antiseptics is a common clinical practice, however, possible cytotoxic effects of these agents may limit their use [22].

According to the outcome of our research, previous studies reported that the routinely clinically used antiseptic PVP-I negatively impacts the viability of various cell types in vitro. Cell death was described for human alveolar bone cells and bovine chondrocytes after short-term incubation with 5% PVP-I [9, 23]. Additionally, exposure of human skin fibroblasts to 0.1% PVP-I completely inhibited cell growth, and 1% solution caused cell death in the majority of the population [24]. Furthermore, Liu et al. [25] reported a cell survival rate of fibroblasts, osteoblasts, and myoblasts of less than 6% exposed to PVP-I concentrations of 0.1% or greater. Similar results were presented in another in vitro study with human chondrocytes [26]. These studies corroborate the findings of our research that PVP-I even at low concentration and short incubation times significantly impacts the cell viability negatively.

Hydrogen peroxide is another commonly used wound antiseptic for which the cytotoxic effect has been proven for various cell types too. For fibroblasts and chondrocytes, a significant reduction of cell viability after incubation with 1.5% H_2_O_2_ for 1 min has been demonstrated [6, 27]. According to our results, the negative effect of hydrogen peroxide on chondrocytes has been shown by Lo et al. [28] even for concentrations considerably lower than those used in our study. Roehner et al. [8] underpin our results reporting a significant decrease in viable chondrocytes after incubation with 3% H_2_O_2_ solution for 30 min.

The notable tolerability of NCT has already been confirmed in several studies. Wirleitner et al. [29] demonstrated that NCT in concentrations of 0.05–1.1 mM did not significantly alter the viability of human peripheral blood mononuclear cells. The viability of dendritic cells treated with NCT in a range of 0.05–0.5 mM for 2 h, analog to the concentrations tested in our study, does not differ from untreated control cells [30]. The findings of these studies are by our results affirming that NCT in low millimole and micromole ranges is well tolerated by chondrocytes in vitro. Furthermore, Nagl et al. demonstrated that an NCT-concentration of 0.01% in vitro did not lead to a decrease in human epidermoid carcinoma cells, but in the presence of 1% NCT cells was impacted negatively, whereas in vivo the concentration of 1% NCT was well tolerated by patients [31]. This difference is not surprising, since it is already known that the in vivo tolerability of NCT is up to 100-fold higher than in vitro [31, 32]. The remarkable tolerability of NCT up to 1–2% was confirmed by investigations in healthy rabbits and human eyes and seemed to be a very well-tolerated and highly effective medication in external otitis and urinary tract infections [20, 33, 34].

The bactericidal effect in combination with the ability of NCT to reduce pro-inflammatory mediators and reactive oxygen species, in concentrations similar to those used in our study, is crucial for the treatment of chronic periodontitis, a disease with a significant negative impact on patient quality of life [19]. Various studies including clinical phase I and IIa studies reported an antimicrobial effect of 1% NCT [17, 21, 31]. Additionally, the antimicrobial effect of NCT on bacterial strains including *S. aureus* has been proven earlier for concentrations in micromole ranges [15, 35]. Nagl et al. reported a 1.9 log^10^ reduction of *S. aureus* at 30 µM NCT-concentration after six hours of incubation [16], a concentration that would be tolerated by chondrocytes as shown in our study.

In our study, we could demonstrate for the first time, that NCT has a less cytotoxic effect on chondrocytes compared to the frequently used antiseptics PVP-I and H_2_O_2_, for which their negative impact on human chondrocytes in vitro has already been reported [6, 26].

Due to a limited proliferation capacity and trend to dedifferentiate in monolayer culture, long-term culturing of primary chondrocytes is challenging. Various studies confirmed that three-dimensional (3D) scaffolds consisting of e.g. collagen, Matrigel, alginate, and agarose hydrogels, are capable of preserving the phenotype of chondrocytes by imitating the extracellular matrix [36]. A 3D cartilage model is a possible next step to observe the tolerability of chondrocytes in a matrix model to underpin the promising result of our monolayer study.

## Conclusion

Application of local antiseptics for the treatment of septic joint infections is common in clinical practice, however, their use may be limited because of possible cytotoxic effects of these agents [22]. In addition to the results of various in-vitro and in vivo studies proving favorable tolerability of NCT in different tissues [20, 21], our results demonstrate that NCT is well tolerated by cartilage cells too. Taking into account that tissue in vivo usually tolerates concentrations many times higher than in vitro [24], the results of our study provide an indication that cartilage in vivo might tolerate a 1% NCT solution, which is already widely used in clinical studies [19].

In combination with the broad-spectrum bactericidal activity, NCT could be conceived as a promising antiseptic for the treatment of septic joint infections, but further studies must be performed to confirm our results.

## Data Availability

All datasets presented in this manuscript are available upon reasonable request from the corresponding author.

## Abbreviations

DMEM/F-12: Dulbecco’s Modified Eagle Medium/Nutrient Mixture F-12
FCS: Fetal calf serum
H_2_O_2_: Hydrogen peroxide
HBSS: Hank’s balanced salt solution
NCT: N-Chlorotaurine
OCT: Octenidine-dihydrochloride
PBS: Phosphate-buffered saline
PHMB: Polyhexanide
PVP-I: Povidone-iodine
3D: Three-dimensional
